# Ex vivo characterization of Breg cells in patients with chronic Chagas disease

**DOI:** 10.1038/s41598-021-84765-x

**Published:** 2021-03-09

**Authors:** Magalí C. Girard, Gonzalo R. Acevedo, Micaela S. Ossowski, Marisa Fernández, Yolanda Hernández, Raúl Chadi, Karina A. Gómez

**Affiliations:** 1 Laboratorio de Inmunología de las Infecciones por Tripanosomátidos, Instituto de Investigaciones en Ingeniería Genética y Biología Molecular “Dr. Hector N. Torres” (INGEBI-CONICET), Buenos Aires, Argentina; 2 Instituto Nacional de Parasitología “Dr. Mario Fatala Chabén”, Buenos Aires, Argentina; 3 Hospital Nacional de Agudos “Dr. Ignacio Pirovano”, Buenos Aires, Argentina

**Keywords:** Infectious diseases, Parasitology, Parasitic infection, Chronic inflammation

## Abstract

Despite the growing importance of the regulatory function of B cells in many infectious diseases, their immunosuppressive role remains elusive in chronic Chagas disease (CCD). Here, we studied the proportion of different B cell subsets and their capacity to secrete IL-10 *ex vivo* in peripheral blood from patients with or without CCD cardiomyopathy. First, we immunophenotyped peripheral blood mononuclear cells from patients according to the expression of markers CD19, CD24, CD38 and CD27 and we showed an expansion of total B cell and transitional CD24^high^CD38^high^ B cell subsets in CCD patients with cardiac involvement compared to non-infected donors. Although no differences were observed in the frequency of total IL-10 producing B cells (B10) among the groups, CCD patients with cardiac involvement showed an increased proportion of naïve B10 cells and a tendency to a higher frequency of transitional B10 cells compared to non-infected donors. Our research demonstrates that transitional B cells are greatly expanded in patients with the cardiac form of CCD and these cells retain the ability to secrete IL-10. These findings provide insight into the phenotypic distribution of regulatory B cells in CCD, an important step towards new strategies to prevent cardiomyopathy associated with *T. cruzi* infection.

## Introduction

Chagas disease, a serious health problem caused by the infection with the protozoan parasite *Trypanosoma cruzi*, comprises an acute and a chronic phase. During the latter, the disease may remain without any detectable symptoms for several decades, or progress toward cardiac or digestive pathological forms, or even a combination of these alterations^1^. The absence of any symptoms, as well as the inflammatory mechanisms leading to tissue damage are attributed mainly to the immune response developed by the host against the parasite, and to the different means by which the parasite avoids it and persists. Although there is a bias towards a predominant pro-inflammatory environment in cardiac patients, while an anti-inflammatory response would prevail in patients without clinical symptoms, data are not completely conclusive^2–4^. In this context, the regulatory mechanisms that tend to return the immune response to a favorable equilibrium could play a relevant role in the context of Chagas disease.

Regulatory B (Breg) cells are a specific B cell subset allocated to restrain the excessive inflammatory response accomplished in different immune-related pathologies, such as autoimmune and allergic diseases, malignancies, infections and solid organ transplantation^5,6^. Through IL-10 production, Breg cells have the capacity to suppress the function and proliferation of Th1, Th17 and follicular helper T (T_FH_) cells, increase polarization of T cells towards the regulatory T (Treg) cell profile, repress the innate response by acting on antigen-presenting cells (dendritic cells, macrophages) and natural killer (NK and NKT) cells, decrease the production of IgG while inducing class switch towards IgG4, among other effects^7^. In addition, Breg cells have also been shown to contribute to immune homeostasis by IL-10-independent mechanisms^8^. However, and due to the absence of subset-specific membrane markers or transcription factors, IL-10 secretion is still the hallmark feature used to distinguish Breg from the rest of the B cells^9,10^. In humans, different B cell lineages that secrete this cytokine and have regulatory functions have been described, such as CD19^+^CD24^high^CD38^high^^11^, CD19^+^CD24^high^CD27^+^^12^, CD19^+^CD5^+^CD1d^+^^13,14^, CD19^+^Tim-1^+^^15,16^ and CD19^+^CD25^+^CD71^+^CD73^−^ cells^7^, depending on the stimulation conditions and the markers used to identify them. Nowadays, the majority of the studies in human samples pinpoint the CD19^+^CD24^high^CD38^high^, the phenotypic signature of the immature transitional B cell population, as the most representative of Breg cells^17^.

In the context of Chagas disease, B cells and their role as antibody secreting cells were amongst the first and most widely studied components of immunity against *T. cruzi*, but little is known about their relevance as antigen presenting cells, cytokine producers and immune modulators^3,18^. To date, Fares et al*.* described that patients with chronic Chagas disease (CCD) have an increased frequency of IL-10- and TGF-β-producing B cells in peripheral blood, both in basal state and upon in vitro stimulation with parasite lysate, hinting that these cells participate in the delicate balance between protection and pathogenesis^19^.

Given that Breg cells have the ability to ameliorate exacerbated inflammatory responses, hampering the development of tissue damage while contributing to pathogen persistence, we sought to analyze *ex vivo* whether changes in the frequency or phenotypic distribution of B and B10 cells from peripheral blood are associated with clinical outcome of patients with CCD.

## Participants and methods

### Subjects included and blood sample collection

Venous blood from patients with CCD and from non-infected donors was drawn into EDTA anticoagulated tubes (BD Vacutainer). Serology for Chagas’ disease was determined by indirect immunofluorescence, enzyme-linked immunosorbent assay (ELISA) or indirect hemagglutination; subjects who had at least two of three positive tests were considered to be infected. After a complete clinical and cardiologic examination covering medical history, physical examination, electrocardiogram (ECG), chest radiography, and echocardiophaphy, patients were stratified according to a modified version of the Kuschnir classification^20^ as follow: Group 0, patients without demonstrable cardiac pathology (G0, n = 10) and Group 1, patients with cardiac involvement (G1, n = 10). Subjects in Group 0 (Kuschnir 0 or K0) had a normal ECG and normal chest radiography, whereas individuals within Group 1 had cardiac alterations such as right and/or left branch blockage and different degrees of conductive functional alterations (Kuschnir 1 or K1, n = 8), left ventricle enlargement (Kuschnir 2 or K2, n = 1) or heart failure symptoms (Kuschnir 3 or K3, n = 1). Because of sample availability reasons, most of the donors included in the G1 belonged to K1 stage. A random group of non-infected donors with negative serology for *T. cruzi* infection were included as the control group (NI, n = 9). The three groups were age- and gender-matched and their clinical features are detailed in Table 1. The exclusion criteria included record of history of treatment with benznidazole or nifurtimox and presence of systemic arterial hypertension, diabetes mellitus, thyroid dysfunction, renal insufficiency, chronic obstructive pulmonary disease, hydroelectrolytic disorders, alcoholism, history suggesting coronary artery obstruction, rheumatic disease, and the impossibility of undergoing the examinations.

**Table 1:** Demographic and clinical features of the study population.

Characteristics	NI (non *T. cruzi*-infected, n = 9)	G0 (CCD without cardiac involvement, n = 10)	G1 (CCD with cardiac involvement, n = 10)
Age,median (interquartile range)	43 (36.5–60)	58 (51.5–61)	56.5 (51.7–64)
Gender, F/M	6/3	5/5	6/4
Kuschnir stage, 0–1–2–3	NA	10–0-0–0	0–8-1–1
PCR, +/−	NA	0/10	1/9
Anti-*T. cruzi* IgG titer, median (interquartile range)	18 (7–31)	14,267 (5,635–42,503)	6,084 (2,070–20,049)

All patients and non-infected donors were age- and gender- matched and clinical features are detailed. The titer of total anti-*T. cruzi* IgG antibodies was consider as the OD_50_ sera dilution factor. *NA* not applicable.

An aliquot of whole blood (4 ml) from each participant was separated and centrifuged for 15 min at 800 g to obtain the plasma, which was stored at − 20 °C until use. The remainder of each sample was used to isolate peripheral blood mononuclear cells (PBMC), as described below. The titer of total anti -*T. cruzi* IgG antibodies in the plasma of patients was determined as previously described^21^. For parasite load, total DNA was extracted from whole blood aliquots using the High Pure PCR Template Preparation kit (Roche Diagnostics Corp., Indiana, USA), following manufacturer-provided instructions.

### Isolation and culture of PBMC

PBMC were isolated from whole blood by Ficoll-Hypaque density gradient centrifugation (GE Healthcare Bio-Sciences AB, Uppsala, Sweden) according to manufacturer-provided instructions, within 4 h after collection. Isolated PBMC were resuspended in fetal bovine serum (FBS, Natocor, Córdoba, Argentina) containing 10% dimethylsulphoxide and cryopreserved in liquid nitrogen until used. The number and viability of thawed PBMC were determined by Trypan blue exclusion staining and only samples with viability higher than 85% were used. Cell suspensions were seeded in 48-well flat-bottom plates at a density of 2 × 10^6^ cells/well in 500 µl of RPMI-1640 medium supplemented with 100 U/ml penicillin, 100 µg/ml streptomycin, 2 mM l-glutamine and 10% heat-inactivated fetal bovine serum (FBS) at 37 °C in a humidified 5% CO_2_ incubator. After 18 h of culture, cells were incubated for additional 5 h with 50 ng/ml Phorbol-12-myristate-13-acetate (PMA) (InvivoGen, San Diego, CA, USA) and 1 µg/ml Ionomycin (IONO) (MP Biomedicals, Santa Ana, CA, USA) or with culture medium only, in the presence of 5 µg/ml Brefeldin A (Biolegend, San Diego, CA, USA) (PIB) before being submitted to the flow cytometry staining process.

### Flow cytometry staining

Cells were transferred to a 96-well V-bottom plate and washed once with PBS by centrifugation at 700 g for 3 min at room temperature (RT). Supernatants were discarded and cells were resuspended in 25 µl of staining solution containing the antibodies detailed in Table 2, diluted in 1X live/dead fixable viability dye (Zombie-Aqua, Biolegend) and incubated 30 min at RT in the dark. After surface markers staining, cells were washed with PBS, fixed with Fixation Buffer (Biolegend) for 20 min at RT in the dark and washed with PBS by centrifugation at 700*g* for 3 min at RT. For intracellular IL-10 detection, cells were permeabilized with Perm-Wash Buffer (Biolegend), stained with anti-IL-10-PE antibody (Table 2) and fixed again with Fixation Buffer. “Fluorescence-minus-one” (FMO) controls were used to determine the cut point for the IL-10 staining, and isotype control staining was considered for cell surface markers gate setting. All antibodies were used at optimal concentrations determined by previous titration experiments. A minimum of 2 × 10^5^ events within the lymphocyte population were acquired in a FACSCanto II (BD Biosciences) flow cytometer using FACS Diva Software (BD Biosciences). Manual analysis of flow cytometry data was carried out with the program FlowJo version 10 (FlowJo LLC, Ashland, OR, USA). The gating strategy used to identify total lymphocytes population, B cells and B10 cells is detailed in Supplementary Figure S1 and subsequent gatings are described in each Figure.

**Table 2:** Fluorescent-labeled antibodies and isotype controls used for flow cytometry experiments.

Antibody/isotype control	Clone	Vendor
BV421-conjugated anti-CD3	UCHT1	Biolegend
PECy5-conjugated anti-CD4	RPA-T4	BD Biosciences
PECy5-conjugated anti-CD19	HIB19	BD Biosciences
APC-conjugated anti-CD27	M-T271	Biolegend
PECy7-conjugated anti-CD24	ML5	Biolegend
APCCy7-conjugated anti-CD38	HB-7	Biolegend
PE-conjugated anti-IL-10	JES3-9D7	Biolegend
BV421-mouse IgG1 ĸ	MOPC-21	Biolegend
PECy5-mouse IgG1 ĸ	MOPC-21	BD Biosciences
APC-mouse IgG1 ĸ	MOPC-21	BD Biosciences
PECy7-mouse IgG 2a ĸ	MOPC-173	Biolegend
APCCy7-mouse IgG1 ĸ	MOPC-21	Biolegend

### Flow cytometry automated analysis

Flow cytometry automated analysis was performed by a density-based data clustering approach, called flowPeaks^22^, implemented in R 3.6.1^23^. Briefly, this method applies k-means clustering across the dimensions used for analysis to summarize the data, and finds density peaks in the distribution of the k-means clusters centroids. Next, the clusters are assigned to the density peak their centroid belongs to, and individual events are tagged with the ID of the peak they fall into. Flow data were filtered within each acquisition batch with flowPeaks, using the scatter parameters, markers CD3 and CD19, and the viability dye, to remove debris, non-viable cells, non-single cells and non-B cell events. After that, normalization across experimental batches was performed using the warp method from the flowStats package^24^. Finally, the filtered and normalized data were pooled into a single matrix, and flowPeaks was applied to it in order to detect B cell subsets with differential expression of markers CD19, CD24, CD27 and CD38. B10 cells were identified using a hard threshold, derived from a density minimum on the distribution of IL-10 expression.

### Statistical analysis

To analyze the differences in frequency of cell populations among groups we applied a generalized linear model (GLM) ^25^ with quasi-binomial distribution of errors and logit link function. Group and phenotype were considered fixed factors and the frequency of cells in each population was the dependent variable. Mean fluorescence intensity data were analyzed by a linear regression model (LM) fitted by maximum likelihood. The group was set as the fixed factor and mean fluorescence intensity was the dependent variable. MFI data were tested for normality and homoscedasticity using Shapiro–Wilk and Bartlett tests, respectively. GLM and LM models were fitted in R 3.6.1^23^. Results are represented with box and whiskers plots showing median value and interquartile ranges. *P *values less than 0.05 were considered statistically significant.

### Ethics statement

The research protocols followed the tenets of the Declaration of Helsinki and were approved by the Medical Ethics Committee of Instituto Nacional de Parasitología “Dr. M. Fatala Chabén” and the Hospital General de Agudos “Dr. Ignacio Pirovano”. All enrolled patients and non-infected donors gave written informed consent, according to the guidelines of the Ethical Committee of each Institution, before blood collection and after the nature of the study was explained.

## Results

### Frequency of total and transitional B lymphocytes is altered in peripheral blood from patients with the cardiac form of chronic Chagas disease

**Figure 1:**
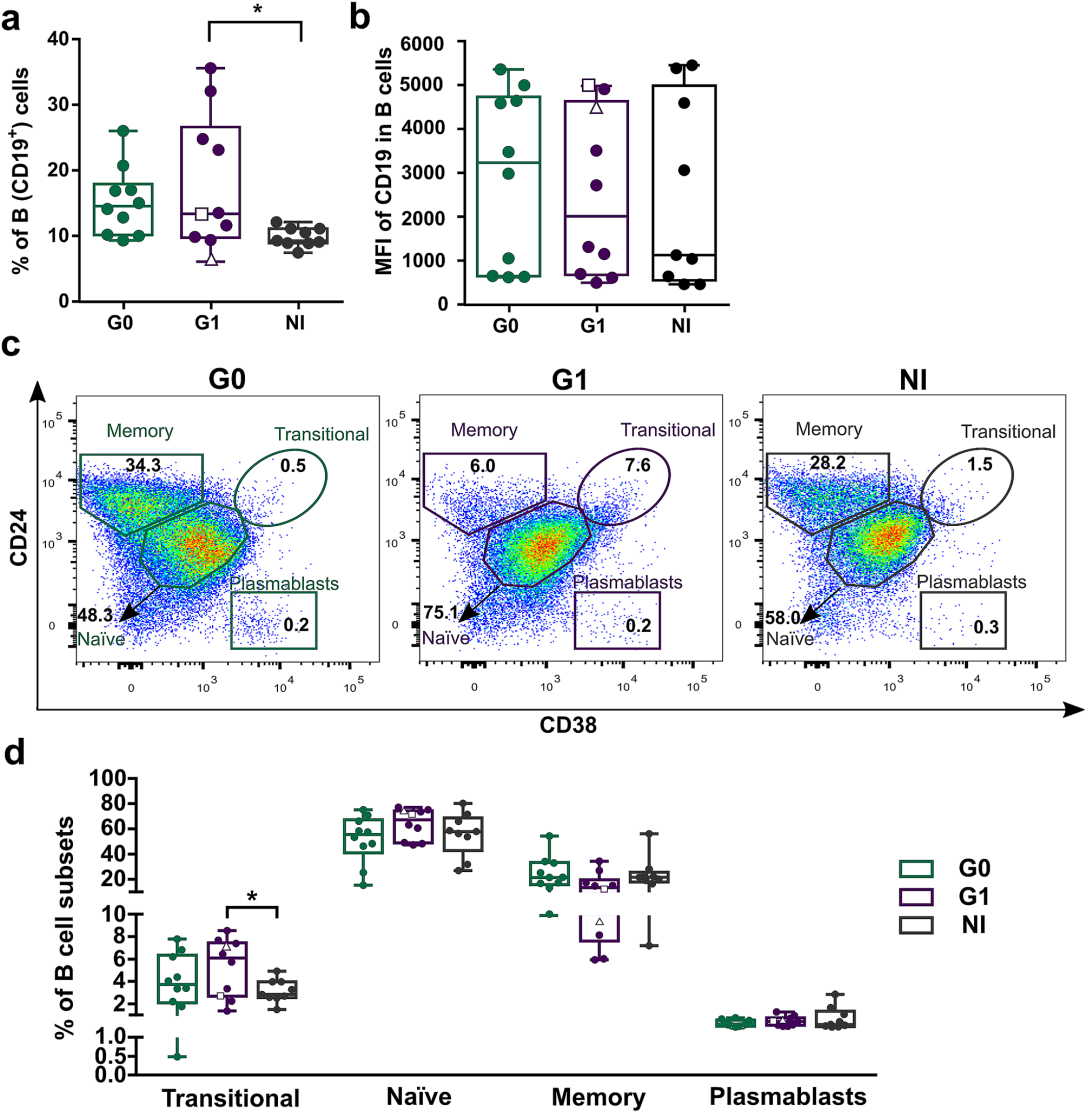
Frequency and phenotypic distribution of total B cells in peripheral blood from chronic Chagas patients and non-infected donors according to CD24 and CD38 expression. (**a,b**) Frequency of B cells (CD3^−^CD19^+^) and mean level of CD19 expression (as mean fluorescence intensity; MFI) in the CD3^−^CD19^+^ population from CCD patients (G0, G1) and non-infected donors (NI). (**c**) Representative dot plots from CCD patients and one non-infected donor, showing the gating strategy used to identify B cell subsets. According to CD24 and CD38 expression levels, B cells were sub-gated into: CD19^+^ CD24^high^CD38^high^ (immature transitional B cells), CD19^+^CD24^int^CD38^int^ (mature naïve B cells), CD19^+^CD24^high^CD38^−^ (memory B cells), CD19^+^CD24^low^CD38^high^ (plasmablasts). (**d**) Frequency of different B cell subsets in total B cells from CCD patients (G0, G1) and non-infected donors (NI). Each dot represents data from one subject; inside G1 group, solid circles correspond to K1 patients, hollow triangle to K2 patient and hollow square to K3 patient. Boxes and whiskers show median value and interquartile range. Statistically significant differences among the groups are indicated (*p < 0.05).

In humans, four different subsets of peripheral blood B cells can be identified according to the expression levels of surface markers CD24 and CD38: CD19^+^CD24^high^CD38^high^ (immature transitional B cells), CD19^+^CD24^int^CD38^int^ (primarily mature naïve B cells), CD19^+^CD24^high^CD38^low^ (primarily memory B cells) and CD19^+^CD24^low^CD38^high^ (plasmablasts)^26,27^. Since the combined expression of these markers has not been addressed in the context of chronic *T. cruzi* infection, we decided to evaluate the frequency of the subsets mentioned above within total CD19^+^ B cells in PBMC from CCD patients without cardiac compromise (G0, n = 10), CCD patients with cardiac involvement (G1, n = 10) and non-infected donors (NI, n = 9). First, we analyzed the frequency of total B lymphocytes and mean fluorescence intensity (MFI) for CD19 in the CD19^+^ cell population. The gating strategy used to identify total B cells (CD3^−^CD19^+^ cells) is illustrated in Supplementary Figure S1. Results revealed a higher percentage of CD19^+^ B cells in patients with cardiac involvement compared to non-infected donors (*p* = 0.016; Fig. 1a). No statistically significant differences were observed between G0 and NI or between CCD groups. Furthermore, similar MFI levels were found across the cohort, independently of the clinical status of the subjects (Fig. 1b). Next, using the gating strategy represented in Fig. 1c, we quantified the frequencies of different B cell subsets according to CD24 and CD38 expression in CCD patients and NI group. Results showed that the frequency of transitional B cells (CD24^high^CD38^high^) within total B cells was higher in patients with cardiac involvement than in non-infected donors (*p* = 0.038). In addition, no significant differences were observed in the frequencies of the other B cell subpopulations among the groups (Fig. 1d).

**Figure 2:**
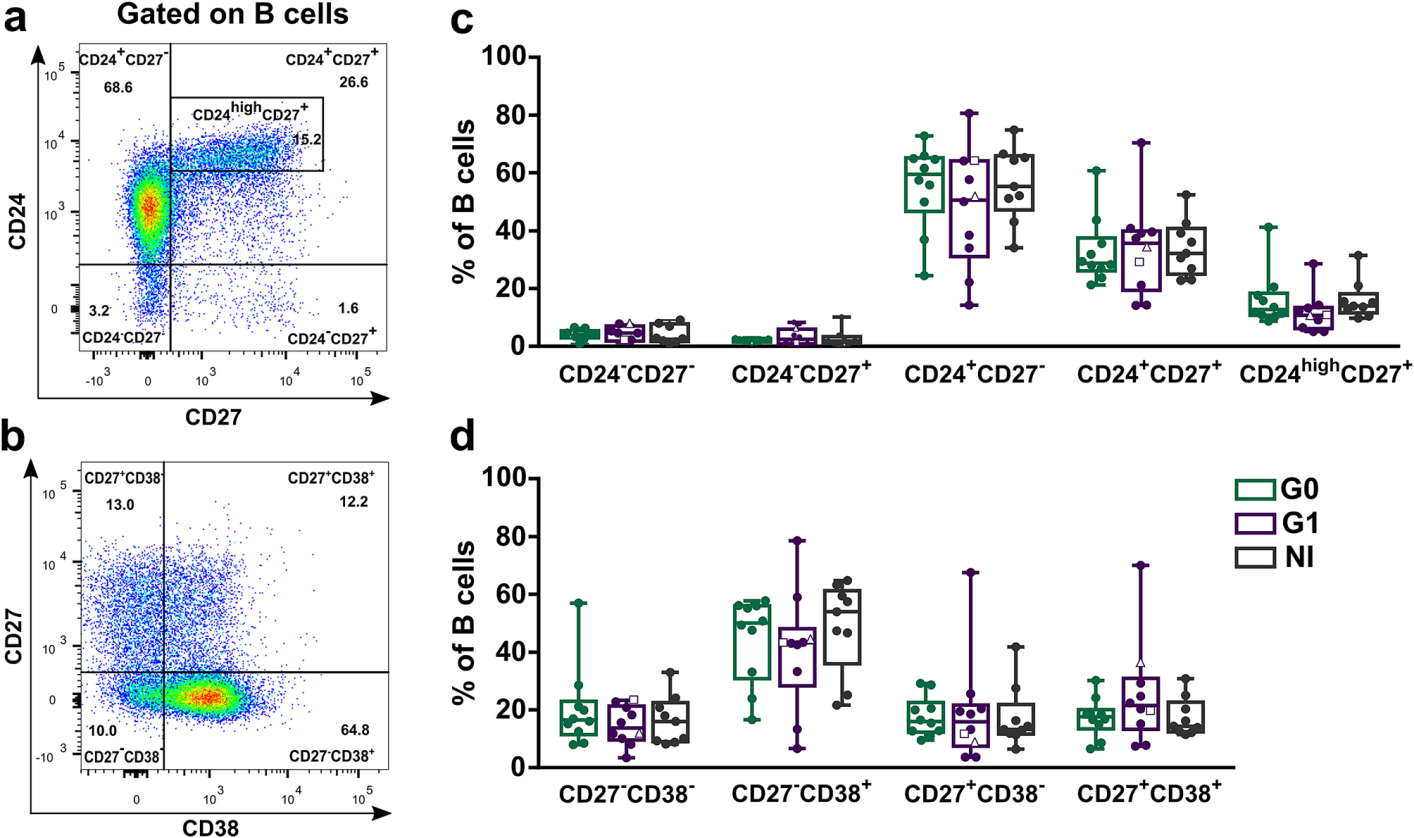
Frequency of B cell subsets based on CD24 and CD27 or CD27 and CD38 markers expression in peripheral blood from CCD patients and non-infected donors. (**a**,**b**) Representative dot plots from a non-infected donor showing the gating strategy used to determine the subsets within total B cells according to the combined expression of CD24 and CD27 or CD27 and CD38. (**c**,**d**) Frequency of B cell subsets in CCD patients (G0, G1) and non-infected donors (NI). Each dot represents data from one subject; inside G1 group, solid circles correspond to K1 patients, hollow triangle to K2 patient and hollow square to K3 patient. Boxes and whiskers show median value and interquartile range.

We also determined the frequency of B cell subsets according to the expression of markers CD24 and CD27 or CD27 and CD38. The gating strategy used to define each cell subset is illustrated for a representative donor in Fig. 2a,b. No differences among the groups were detected in any of the subpopulations evaluated (Fig. 2c,d).

### Phenotypic distribution but not frequency of the B10 cell compartment is changed in peripheral blood from patients with the cardiac form of CCD

**Figure 3:**
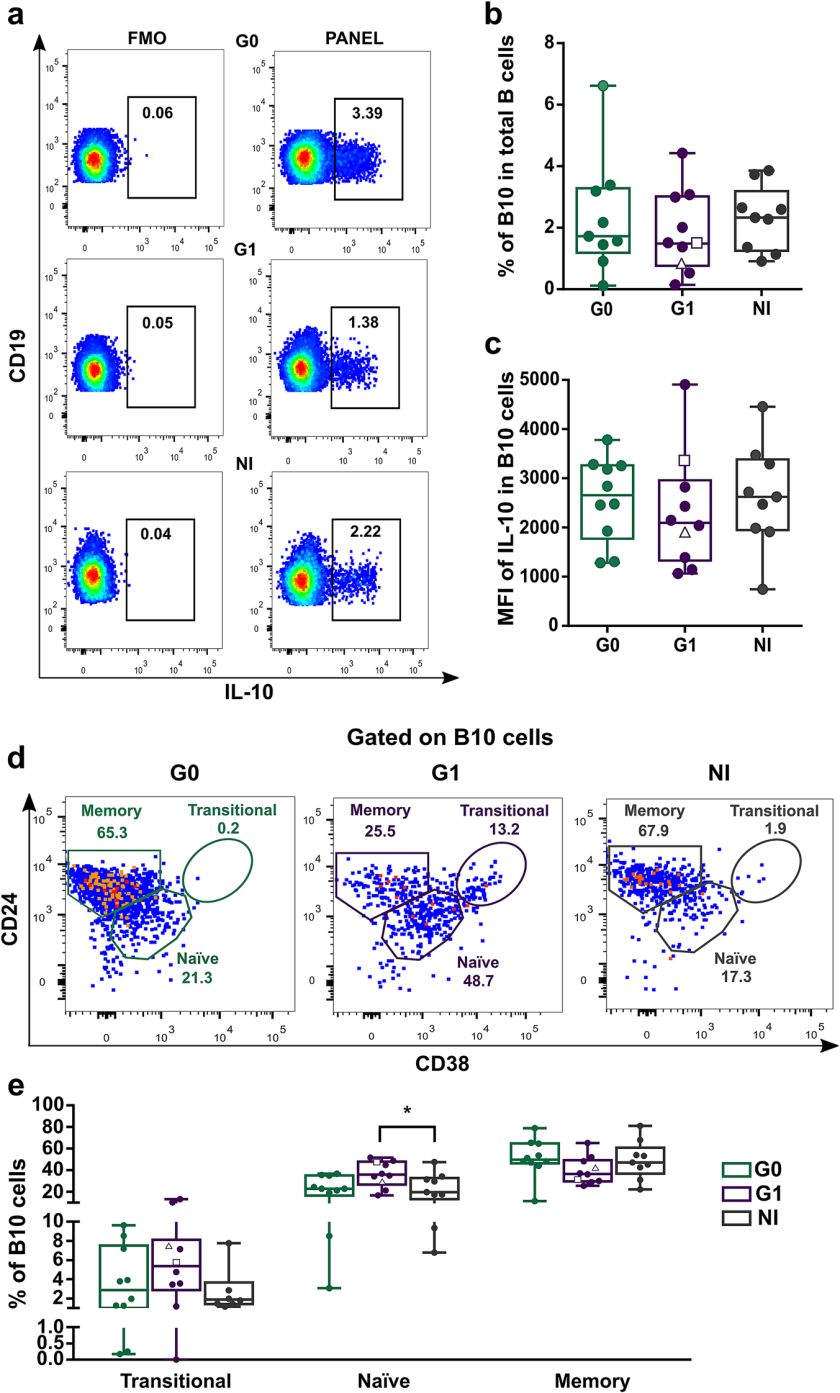
Frequency and phenotypic distribution of B10 cells according to CD24 and CD38 expression in peripheral blood from CCD patients and non-infected donors. (**a**) Representative dot plots showing the gating strategy used to determine the IL-10^+^ B cells (B10 cells) in PIB stimulated PBMC. Fluorescence minus one controls (FMO) were used to define the negative populations for each sample. (**b**,**c**) Frequency of B10 cells in total B cells and mean fluorescence intensity (MFI) for IL-10^+^ in B10 cells in CCD patients (G0, G1) and non-infected donors (NI). (**d**) Representative dot plots showing the gating strategy used. IL-10 expressing B-cells (B10 cells) were gated according to subject- and condition-matched FMO control tubes and were further sub-gated using CD24 and CD38 to identify the phenotypical distribution of these populations. (**e**) Frequency distribution of B10 cells according to CD24 and CD38 expression in CCD patients (G0, G1) and non-infected donors (NI). Each dot represents data from one subject; inside G1 group, solid circles correspond to K1 patients, hollow triangle to K2 patient and hollow square to K3 patient. Boxes and whiskers show median value and interquartile range. Statistically significant differences among groups are indicated (*p < 0.05).

Since the CD24^high^CD38^high^ transitional B cell population has been associated with IL-10-mediated, regulatory B cell functions in human autoimmune and infectious diseases^11,28,29^, we next studied IL-10 producing B cells (B10 cells) in peripheral blood from patients with the different clinical forms of CCD. We assessed the frequency of total B10 cells and B10 cell subsets according to CD24, CD38 and CD27 expression^11,29^. The gating strategy is shown in Supplementary Figure S1 and Fig. 3a. Results showed that G1 patients have a tendency, although non-statistically significant, to a decreased frequency of B10 cells compared to non-infected donors (Fig. 3b). Furthermore, no differences in MFI of IL-10 in IL-10^+^ B cells were observed among the groups (Fig. 3c).

The analysis of phenotypic distribution of B10 cells revealed that in G1 patients the frequencies of B10 cells with transitional and naïve phenotype were higher compared to those from NI donors (Fig. 3d). However, only the frequency of naïve B10 cells was statistically different among the groups (*p* = 0.036; Fig. 3e). This augmentation was related to a decrease in the frequency of B10 cells with memory phenotype in G1 patients (Fig. 3e). In fact, when we analyzed the ratio between both subpopulations in this group, we observed a shift towards naïve B10 cells that was undetectable in G0 patients and in non-infected individuals (ratio naïve/memory B10 cells in G1 = 0.98; G0 = 0.46; NI = 0.42).

**Figure 4:**
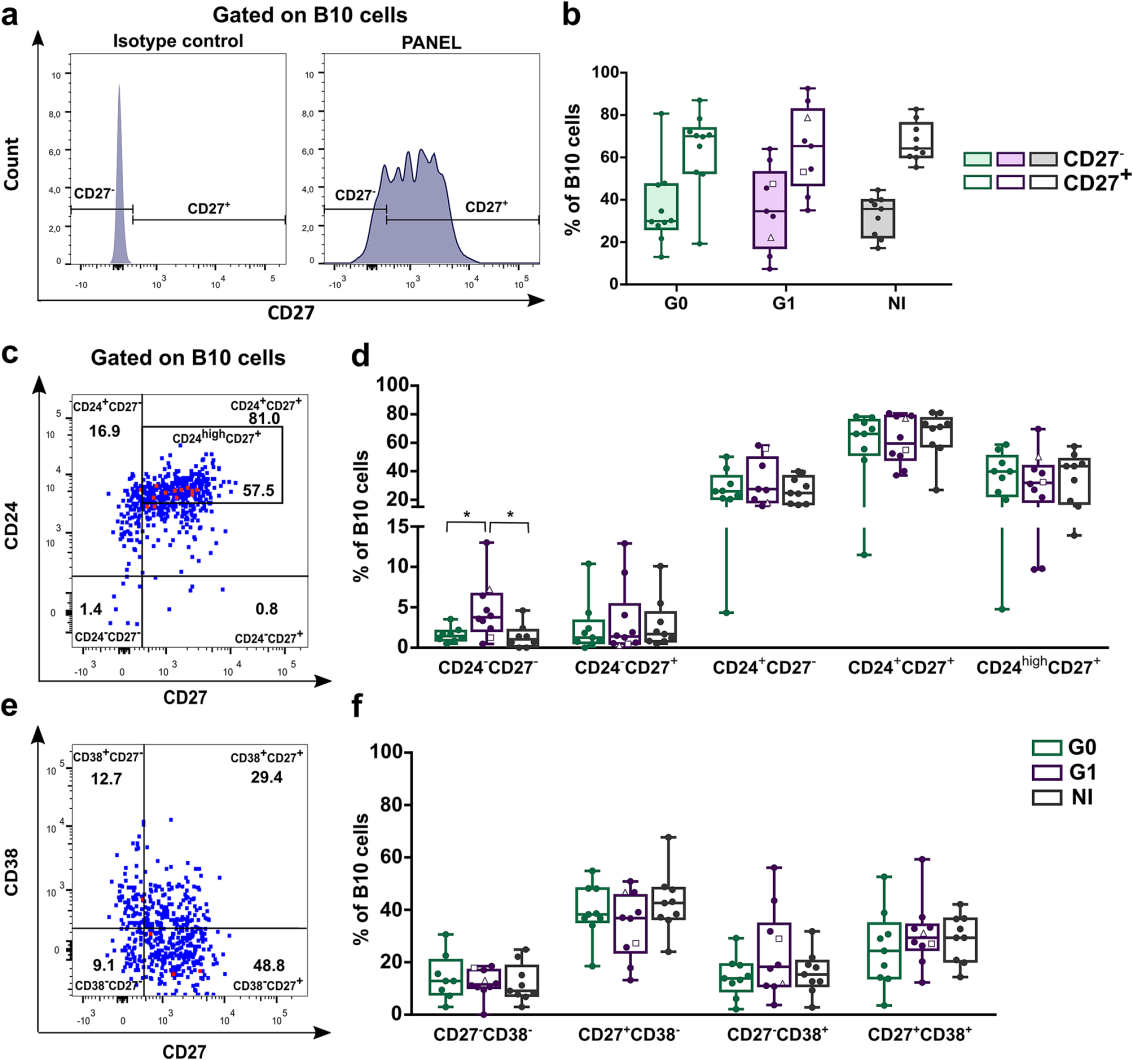
Phenotypic distribution of B10 cells according to CD27, CD27-CD24 and CD27-CD38 expression in CCD patients and non-infected donors. (**a**) Histograms showing the gating strategy used to determine CD27^−^ (naïve) and CD27^+^ (memory) populations within B10 cells in PIB stimulated PBMC. One representative non-infected donor is illustrated. (**b**) Frequency of CD27^−^ and CD27^+^ B10 cells in CCD patients (G0, G1) and non-infected donors (NI). (**c**,**d**) Representative dot plots from a non-infected donor showing gating strategy used to determine the subsets within total B10 cells based on the combine expression of CD24 and CD27 or CD27 and CD38 in PIB stimulated PBMC. (**e**,**f**) Frequency of B10 cell subsets within CD24-CD27 and CD27-CD38 subpopulations in CCD patients (G0 G1) and non-infected donors (NI). Each dot represents data from one subject; inside G1 group, solid circles correspond to K1 patients, hollow triangle to K2 patient and hollow square to K3 patient. Boxes and whiskers show median value and interquartile range. Statistically significant differences among groups are indicated (*p < 0.05).

Since CD27 is a memory marker of human B cells^30^, we evaluated the frequency of B10 cells according to CD27 expression alone and in combination with CD24 and CD38. Results showed that B10 cells were mainly found in the CD27^+^ (memory) population in the non-infected group, as it had been reported in blood samples from healthy donors^29^ and the same pattern was observed in CCD patients (Fig. 4a,b). In addition, there were no differences in the frequency of memory (CD27^+^) and naïve (CD27^−^) B10 cells subsets among the groups (Fig. 4b).

Analysis of B10 cells subsets according to their CD24–CD27 expression showed an enrichment in CD24^−^CD27^−^ cells in G1 compared to G0 and NI donors (*p* = 0.039 and *p* = 0.027 respectively; Fig. 4c,d). Frequencies of B10 cell subsets according to CD27–CD38 expression did not shown statistically significant differences among the groups (Fig. 4e,f).

**Figure 5:**
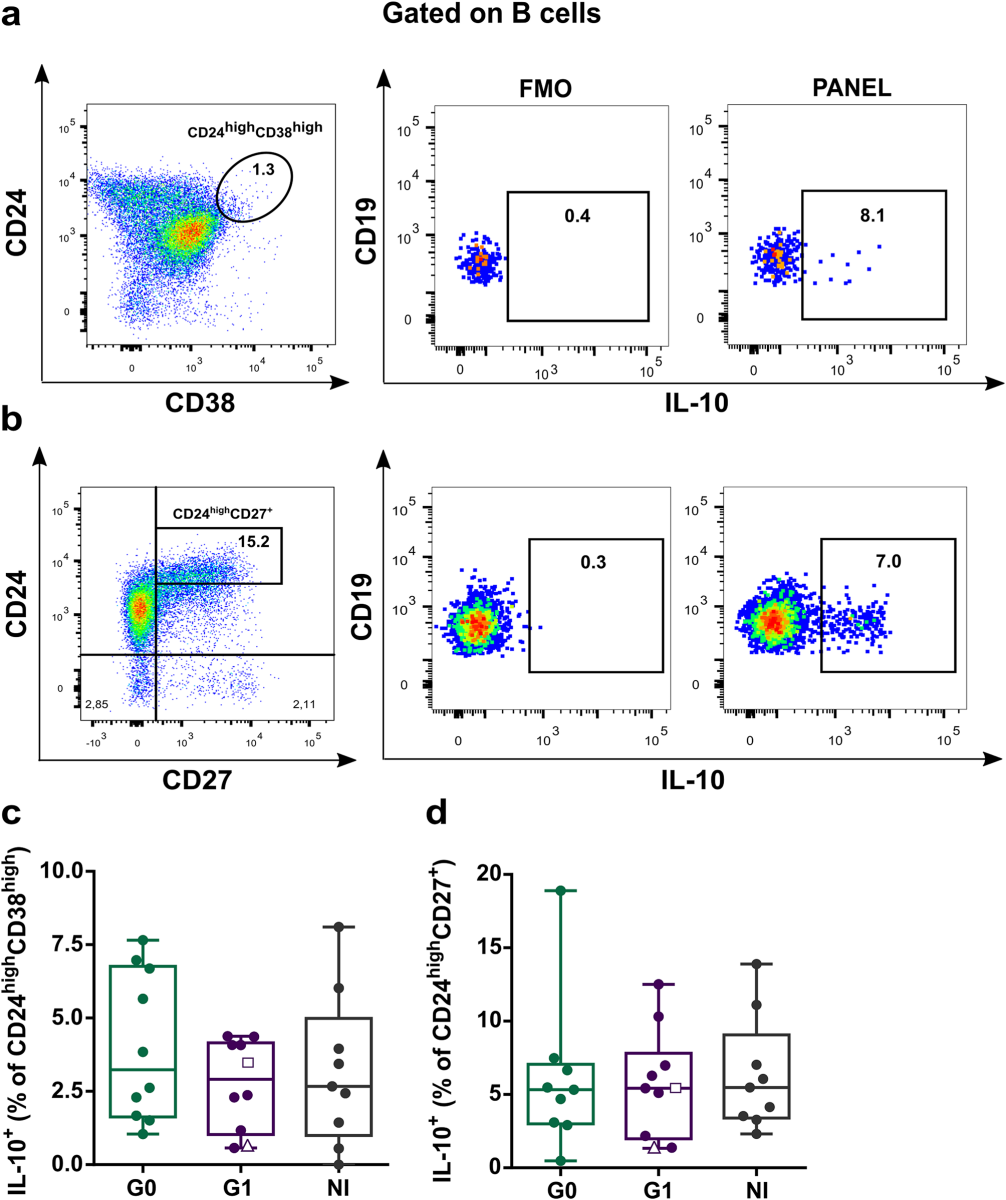
IL-10 producing cells within CD24^high^CD38^high^ transitional and CD24^high^CD27^+^ memory B cell populations in CCD patients and non-infected donors. (**a**,**b**) Representative dot plots showing the gating strategy used to determine frequency of IL-10^+^ cells in CD24^high^CD38^high^ and CD24^high^CD27^+^ B cells. (**c**,**d**) Frequencies of IL-10^+^ cells within transitional (CD24^high^CD38^high^) and memory (CD24^high^CD27^+^) compartments in CCD patients (G0, G1) and non-infected donors (NI). Each dot represents data from one subject; inside G1 group, solid circles correspond to K1 patients, hollow triangle to K2 patient and hollow square to K3 patient. Boxes and whiskers show median value and interquartile range.

Given that IL-10 producing B cells are not confined to a single B cell subset, several studies have tried to determine which B cell populations are enriched in IL-10^+^ cells. In this sense, most of the studies performed in human samples show that CD24^high^CD38^high^ and CD24^high^CD27^+^ compartments exhibit higher frequencies of IL-10 producing cells than their respective counterparts^11,12,29^. Taking this into account, we aimed to evaluate the frequency of IL-10^+^ cells within CD24^high^CD38^high^ and CD24^high^CD27^+^ enriched subpopulations (Fig. 5a,b, respectively). With this approach, we did not find differences between the groups (Fig. 5c,d, respectively).

### Data-driven clustering of B and B10 cells

**Figure 6:**
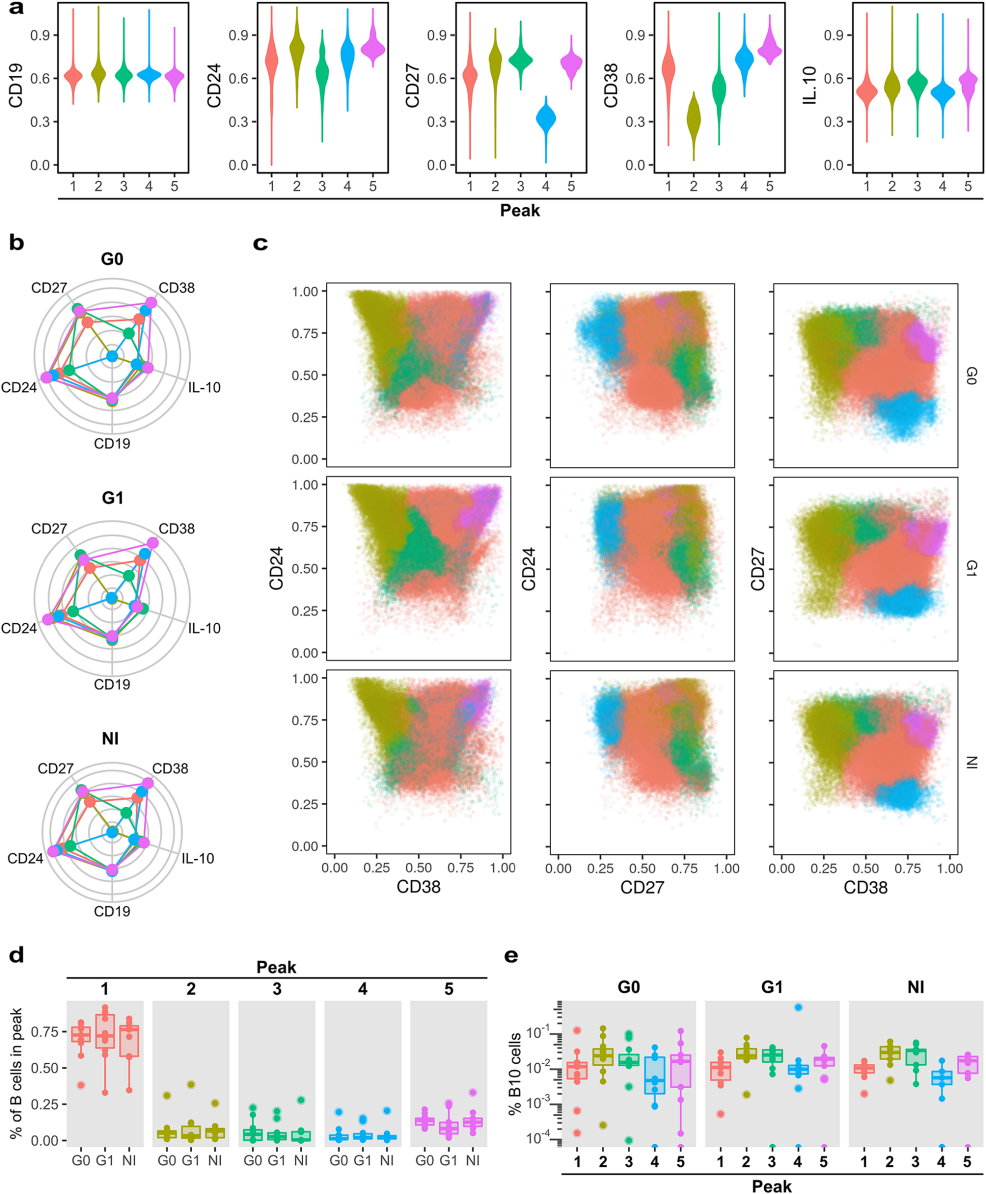
Automatic gating performed by flowPeaks algorithm of B cell subsets based on CD19, CD24, CD27 and CD38 expression. (**a**) Violin plots show the distribution of the expression of each marker for each peak. (**b**) Spider plots display the median expression for each marker in the different peaks. One graph is shown for each group of CCD patients (G0, G1) and non-infected donors (NI). (**c**) Representative biaxial dot plots showing the distribution of peaks based on CD24-CD38, CD24-CD27 and CD27-CD38 expression. (**d**) Box plots showing the percentage of B cells within each peak in G0, G1 and NI groups. (**e**) Box plots indicate the percentage of B10 cells within each peak in the G0, G1 and NI groups.

Next, we applied an unsupervised clustering based approach to characterize in a multiparametric manner, event distributions within B and B10 cells in the context of CCD, and to evaluate the existence of subpopulations that cannot be easily identified by manual gating analysis. For this purpose, we used the flowPeaks method^22^, an automated, density-based and data-oriented clustering approach. We identified five B cell clusters (“peaks”) based on the expression of CD19, CD24, CD27 and CD38, being the latter the marker with the most diverging expression profiles among B these cell subpopulations (Fig. 6a). Conversely, no differences were observed in the expression distribution of CD19 (Fig. 6a,b). Analyzing the features of each cluster, we observed that the peak 1 displayed an intermediate expression level of CD24, CD27 and CD38. Peaks 2 and 3 revealed high expression of CD27, but peak 2 had higher CD24 and lower CD38 levels than peak 3. Peak 4 and especially peak 5 expressed the highest levels of CD24 and CD38. Furthermore, CD27 levels were low in peak 4 and intermediate in peak 5 (Fig. 6a,b).

When we compared the subpopulations identified using flowPeaks with those designated by manual gating, we observed that the region where conventional transitional B cells are located by CD24 and CD38 expression, was populated with cells assigned to peaks 1, 4 and 5. In addition, peak 4 and 5 partially overlapped in non-infected subjects and G0 patients, while G1 patients displayed a spreading of peak 5 into a region of higher CD24 and CD38 expression. The manually gated memory B cell population was mostly represented within peak 2, expressing the characteristic high level of CD27 and CD24 and low expression of CD38. Finally, the manually defined mature naïve B cell population was mainly composed by the events corresponding to peak 3 but also peaks 1 and 4 (Fig. 6c). Memory CD24^high^CD27^+^ B cell subset by manual gating analysis was enclosed within peaks 1, 2, 3 and 5 (Fig. 6c). Next, we analyzed the frequencies of flowPeaks-gated subsets, but no statistically significant differences were found among the groups (Fig. 6d). In addition, peaks 2, 3 and 5 were enriched in B10 cells in all groups (Fig. 6e).

**Figure 7:**
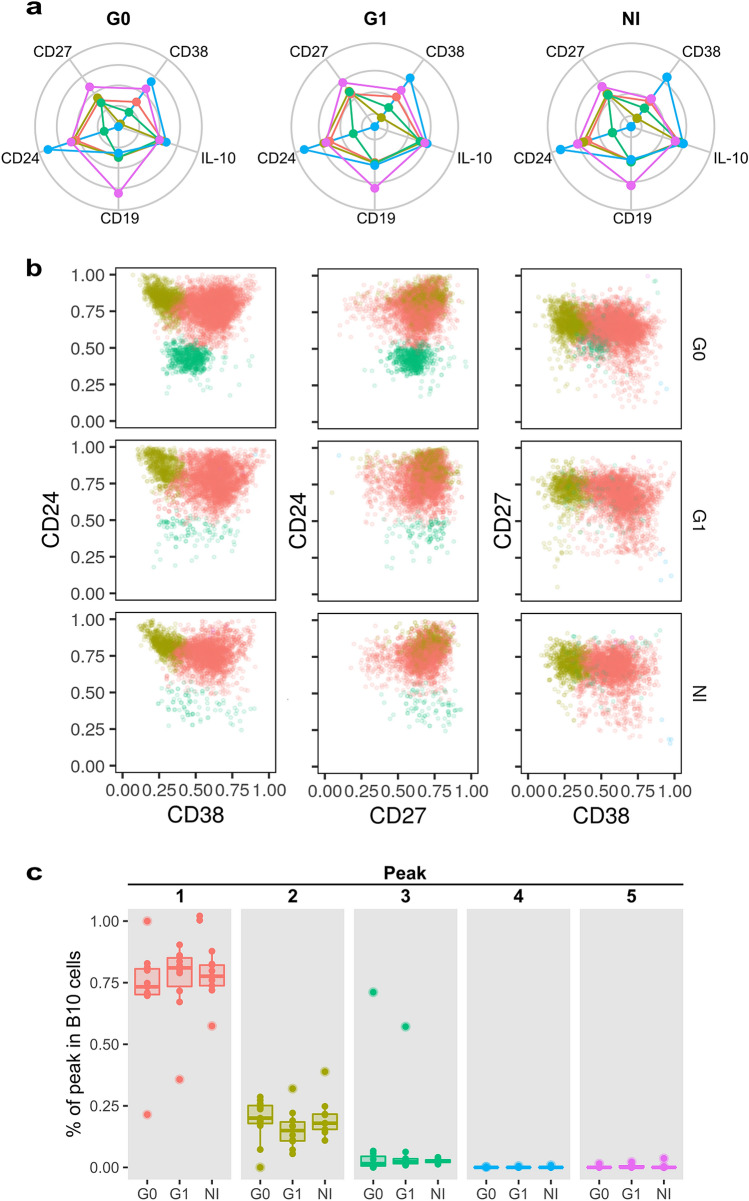
Automatic gating performed by flowPeaks algorithm of B10 cell subsets based on CD19, CD24, CD27 and CD38 expression. (**a**) Spider plots display the median expression for each marker in the different peaks. (**b**) Representative biaxial dot plots showing the distribution of peaks within B10 cells based on CD24–CD38, CD24–CD27 and CD27–CD38 expression. (**c**) Box plots showing the percentage of cells within each peak in B10 cells in G0, G1 and NI groups.

The flowPeaks method was also applied on pre-gated B10 cells, in order to evaluate their phenotypic distribution in terms of the aforementioned markers. Results showed five different density peaks in the data, although two of them contained too few events to sustain any ulterior analysis. Of note, peaks 1–5 from this analysis were not related in any way to peaks 1–5 from the bulk B cell analysis presented in Fig. 6, as they originated from independently run analyses. In the B10 cell subset analysis, peak 1 was characterized by intermediate expression of CD24, CD38 and CD27. Peak 2 included B10 cells with slightly higher levels of CD24 and CD27 but reduced levels of CD38 expression compared to peak 1. Finally, peak 3 contained B10 cells with low levels of CD24 and CD38 but intermediate levels of CD27 (Fig. 7a). Data analysis within each group showed that frequency of B10 cells in peak 1 was higher than B10 cells in peak 2 and 3 (*p* < 0.001; Fig. 7b,c). Comparing among groups, we found that peak 3 had a trend of higher frequency in G0 than G1 patients and non-infected subjects while no differences were observed in the frequency of peaks 1 and 2 (Fig. 7b,c).

## Discussion

In this study, we characterized for the first time the *ex vivo* B cell compartment in patients with chronic Chagas disease with and without cardiac involvement, in terms of their grouping in immature transitional (CD19^+^CD24^high^CD38^high^), primarily mature (CD19^+^CD24^int^CD38^int^), primarily memory (CD19^+^CD24^high^CD38^−^) B cells, and plasmablasts (CD19^+^CD24^−^CD38^high^)^26,27^. We found that immature transitional peripheral blood B cells had higher frequencies in patients with cardiac involvement compared to non-infected subjects, and that this expansion could be conjoined with an increase in the percentage of total CD19^+^ B cells. In a previous work, Fernandez et al*.*^31^ observed an augmentation in the frequency of CD19^+^IgG^+^CD27^−^IgD^−^ “double negative” memory and CD19^+^IgM^+^CD27^−^IgD^+^ transitional/naïve B cells with a selective reduction of their classical memory CD27^+^ counterparts. In addition, and contrariwise to our observation, the frequency of CD19^+^ B cells in infected individuals was significantly lower compared to non-infected subjects. In this regard, and although it would be interesting to correlate our findings with those described therein, it should be noted that B cell subsets were designated based on different surface markers. Furthermore, the infected subjects in the cohort of the mentioned study were grouped together for the analysis, independently of their clinical status. However, both studies clearly depict an alteration in the peripheral B cell compartments of infected individuals during the chronic phase of Chagas disease with a predominance of intermediate forms between the immature and mature phenotypes. The expansion of circulating immature transitional with a decrease in memory B cells is a phenomenon that could be due to genetically intrinsic B cell abnormalities as well as extrinsic cellular or molecular factors that regulate B cell lymphopoiesis^32^. In some immunodeficient states and autoimmune processes, transitional B cell alterations are associated with high expression levels of the B-cell activating factor of the TNF family, BAFF or IL-7^27,33–37^, both essential for these cells to outright their development^38–42^. Interestingly, there are evidences that BAFF and myeloid cell derived factors are abundantly secreted early after *T. cruzi* infection, leading not only to interferences in the development of mature B cells but also to a self-reactive response through polyclonal activation^43–46^. What´s more, the IL-7/IL-7R axis was found to be altered in CCD patients with severe cardiomyopathy^47^. Although Albareda et al*.* pointed out the preeminent role of this cytokine in controlling T cell homeostasis^47^, the augmentation of serum levels of IL-7 detected in patients with CCD could be directly linked to our observation in the B cell compartment.

Production of IL-10 is the ultimate feature of several defined B regulatory phenotypes^48^, and the broadest phenotypic marker of human Breg cells described so far^49^. Several subsets of IL-10-producing human B cells have been proposed in studies centered on different diseases, immunological contexts and stimulation conditions. These phenotypes include CD1d^+^CD5^+^^13,14,50,51^, Tim-1^+^^15,16^, immature transitional CD24^hi^CD38^hi^^11,48^ and memory CD24^high^CD27^+^^12^ B cells, the last two being regarded as the main IL-10 producers^12,52^.

Focusing on chronic infections, a potent immunosuppressive role has been reported for B10 cells, acting mainly onto antigen specific CD8^+^ T cells^53^. In HIV-infected children and adolescents, an increased frequency of circulating Breg cells induced by influenza immunization has been linked to a poor response to vaccination^54^. In leishmaniasis, IL-10 secreted by CD5^+^CD1d^+^ B cells polarized the Th cell response toward the Th2 phenotype, leading to susceptibility to infection with the parasite in BALB/c mice^55^. In fact, incubation of human B cells with *Leishmania infantum* amastigotes induced IL-10 production by CD24^+^CD27^−^ B cells, which partially modulated INF-γ and TNF-α secretion by T cells^56^. Although B10 cells are involved in the maintenance of homeostasis in the immune system^5,57^, these findings highlight the importance of microorganism- triggered immunosuppressive mechanisms that could favor their persistence and contribute to poor vaccine response^58,59^. Nonetheless, and given the implications of uncontrolled inflammation in the context of Chagas disease, B10 cells are likely to contribute to the delicate balance between parasite clearance and inflammatory pathogenesis.

As mentioned above, the only prior research addressing the role of B10 cells in chronic Chagas disease was carried out by Fares et al*.*^19^. Therein, *T. cruzi* infected patients presented a slightly higher frequency of IL-10-producing CD19^+^CD1d^+^CD5^+^ cells compared to non-infected subjects. Here, we found that the frequency of B10 cells was similar among the groups, but their phenotypic distribution based on surface markers CD24 and CD38 was altered in patients with cardiac involvement. While no differences were observed in the frequency and phenotypic distribution of B10 cells between non-infected donors and patients without cardiac involvement, those with cardiac manifestations had significantly larger naïve and immature transitional subpopulations within B10 cells. This augmentation seems to be associated with a decrease tendency in the frequency of CD24^high^CD38^low^ memory B10 cells. In fact, we found a change in naïve/memory B10 cell ratio in patients with cardiac alterations, favoring the former phenotype. Naïve and memory B10 cells are thought to have different functions in autoimmune and infectious diseases in humans, and their IL-10 production depends on specific activation signals in a particular immunological environment. While naïve B cells IL-10 production requires CD40 engagement to prevent inflammatory responses and maintain immune homeostasis, memory B cells IL-10 production cells activates by a combined TLR, BCR and CD40 signaling to resolve the adaptive pathogen-specific immune response and autoimmune disease inflammation^60^. It is not surprising that patients in the severe form of the disease display a phenotype towards naïve B10 cells since their immunological status refers an activated immune status with an important autoimmune component^3,61^.

Interestingly, although CD24^high^CD27^+^ IL-10 producing cells seemed not to be altered, the percentage of CD24^−^CD27^−^ cells was also significantly increased in patients with cardiac manifestations. The functional features of this subpopulation remain elusive and the relevance of this augmentation will be a matter of further investigation in our laboratory.

Furthermore, our data revealed no differences in IL-10 production by CD24^high^CD38^high^ and CD24^high^CD27^+^ B cells among the individuals independently of their clinical status, suggesting that B cells from patients with cardiac involvement have a regulatory phenotype mainly due to an expansion of the CD24^high^CD38^high^ immature transitional compartment, independently of their capacity to secrete IL-10. In relation to this, it was established that the frequency of CD24^high^CD38^high^ B cells changes in different clinical scenarios. In autoimmune settings, such as primary Sjögren syndrome and systemic lupus erythematosus (SLE), the frequency of this subset was shown to increase compared to healthy controls, while in HIV infection, an important decrease was noted^62^. Furthermore, a connection between CD24^high^CD38^high^ B cells and immune regulatory function was evident in the favorable clinical outcome of patients with chronic inflammatory and autoimmune diseases^63^. In the context of CCD, our results suggest a relationship between the cardiac manifestation of the disease and alterations in the frequency of transitional B cells.

Chronic Chagas cardiomyopathy and dilated cardiomyopathy (DCM) have a similar structural disarrangement that leads to ventricular dilatation, but the former is characterized by severe myocarditis and a dense fibrosis that surrounds each myocardial fiber or group of myocardial fibers^64^. Conversely to our observation, Guo et al*.* showed that the frequency of CD19^+^IL-10^+^ B cells in peripheral blood from DCM patients was higher than those from healthy individuals under the same stimulation conditions followed in our study^65^. However, when stimulated with CD40L and CpG, the percentage of CD19^+^IL-10^+^ B cells diminished in DCM patients compared to a healthy control group^66^. In addition, the frequency of CD24^high^CD38^high^ transitional B cells and CD24^high^CD27^+^ B cells in DCM patients were comparable with that of healthy individuals, while IL-10 production by CD24^hi^CD27^+^ B cells from DCM patients was decreased. Although the experimental conditions differ between our study and that by Jiao et al. ^66^ and this could account for the discrepancies seen in the populations mentioned above, we cannot rule out that these alterations may be linked to the histopathological findings ascribed to each disease.

Finally, we also applied an automatic clustering based approach to identify the main B and B10 cellular subtypes from our multidimensional flow cytometry dataset. These unsupervised methods have been used previously with multidimensional flow cytometry data, allowing researchers to identify cell subsets, including characterized and novel subpopulations of B cells of unknown biological significance^62,67^. In our study, analysis of the expression of CD19, CD24, CD38 and CD27 parameters was able to identify several subsets with features consistent with the conventional populations previously described in the literature, in the context of healthy or pathological state. Furthermore, flowPeaks had the ability to reveal different expression levels of the memory marker CD27 enclosed within the transitional B cell subset. In fact, memory CD24^high^CD27^+^ regulatory B cells appeared as a heterogeneous population, which also included cells belonging to the transitional B cell compartment, expressing intermediate or high levels of CD27 as part of the events within peak 1 (CD27^int^CD24^high^CD38^int^), peak 2 (CD27^high^CD24^high^CD38^low^) and peak 3 (CD24^low^CD38^low^CD27^high^). Our data agree with a previous report by Simon et al. where several subsets of transitional B cells are described within the CD24^high^CD38^high^ population, including CD27^−^ transitional B cells and a population with an atypical activated memory phenotype, characterized by CD27^+^ expression^62^. It is important to mention that in the aforementioned study, a panel included also CD21, CD32, IgM, IgD, CD5 and CD10 markers was used to distinguish transitional B cell subsets. Considering these findings, it would be interesting to elucidate which subset within the transitional B cells is augmented in patients with the cardiac form of CCD and whether it displays differential abilities to regulate T cell response. In addition, flowPeaks method showed that only patients with cardiac involvement displayed a spreading of the subset that expressed the highest levels of CD24 and CD38 to a region of augmented expression of both markers. Our analysis did not detect any statistically significant differences among the groups under study in the proportion of the B cell subsets identified. It should be acknowledged, however, that the continuous nature of the expression levels of surface markers like CD24 and CD38 on B cells, with no preeminent modes or peaks in their distributions, constrain the power of density-based algorithms like flowPeaks to discern distinct populations. Although this may be perceived as a limitation of this type of approach, it actually hints at a broader, conceptual issue in single-cell level analyses: as useful as it is to partition cell populations into subsets for their characterization, often times this compartmentalization fails to reflect the true heterogeneity of cell phenotypes and functional profiles. This is particularly evident in the case of B cells. Nonetheless, we cannot rule out that the non-statistically significant trends, like the one observed for the B10 cells clustered in peak 3, which seem to have an increased frequency in CCD patients, might become significant if a larger cohort is studied. On that note, increasing the statistical power of our assessment by raising the number of enrolled subjects in each group may result in other non-significant trends observed in the manual gating analysis of our dataset becoming statistically significant as well.

In summary, our work shows that immature transitional CD24^high^CD38^high^ B cells are greatly expanded in patients with the cardiac form of CCD, and that these cells keep the same capacity to secrete IL-10 compared to non-infected donors. Additionally, flowPeaks gating revealed that this augmentation could be related with an increase in CD24 and CD38 expression within transitional B cells. This expansion might be the result of defective B cell development during acute *T. cruzi* infection or could be the consequence of a pro-inflammatory state. Remarkably, the distribution of naïve, transitional and memory B cells inside the B10 cells follows the same pattern in chronic patients without cardiac involvement and non-infected individuals. Finally, our data are insufficient to define whether these immature transitional CD24^high^CD38^high^ B cells or some subset within this population have deficiencies in their regulatory function and correlate with the clinical outcome of patients with chronic Chagas disease. We expect that our ongoing *in vitro* studies aiming at the elucidation of the Breg cells’ impact on CD4^+^ and CD8^+^ T cells may help to address this matter.

## Supplementary Information

Supplementary information.
