# Supplementary material for: Ex vivo characterization of Breg cells in patients with chronic Chagas disease
Source: Sci Rep. 2021 Mar 9;11:5511. doi: 10.1038/s41598-021-84765-x (PMC7943772; doi:10.1038/s41598-021-84765-x)
Supplement: Supplementary file 1 — Supplementary information. [file 41598_2021_84765_MOESM1_ESM.docx]

**Supplementary Figure S1**

*Ex vivo* characterization of Breg cells in patients with chronic Chagas disease

Magalí C. Girard^1^, Gonzalo R. Acevedo^1^, Micaela S. Ossowski^1^, Marisa Fernández^2^, Yolanda Hernández^2^, Raúl Chadi^3^, Karina A. Gómez*^1^.

^1^Laboratorio de Inmunología de las Infecciones por Tripanosomátidos. Instituto de investigaciones en Ingeniería Genética y Biología Molecular “Dr. Hector N. Torres” (INGEBI-CONICET).

^2^Instituto Nacional de Parasitología “Dr. Mario Fatala Chabén”.

^3^Hospital Nacional de Agudos “Dr. Ignacio Pirovano”.

*email: gomez@dna.uba.ar.

**
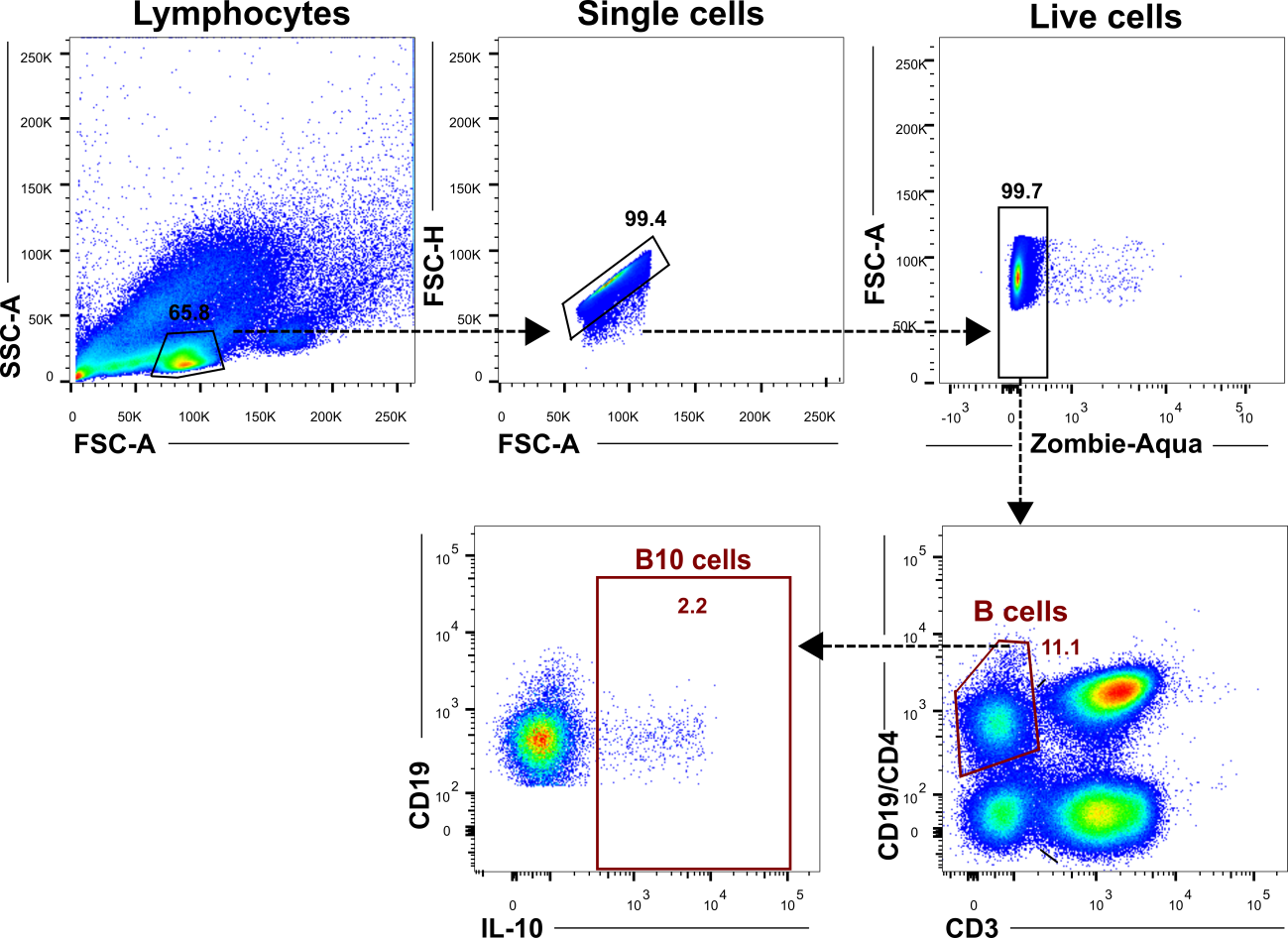
**

**Supplementary Figure S1: General gating strategy used to define B and B10 lymphocyte populations to be studied in CCD patients and non-infected donors.** Representative plots obtained by the manual gating of a sample by flow cytometry, based on the expression of CD3, CD4, CD19 and IL-10 markers. Cells were gated according to forward scatter/side scatter area (FSC-A/SSC-A) criteria to identify the total lymphocytes population. Non-single cell events and cells with compromised viability were excluded by gating on the FSC-A/FSC-H and Zombie Aqua channels, respectively. B-cells were gated using CD19 and CD3 markers (CD3^-^CD19^+^ population). Isotype controls were used to determine cut points for each marker. IL-10^+^ cells were gated according to subject- and condition-matched FMO control tubes. B and B10 cells were further sub-gated according to CD24, CD38 and CD27 markers to identify the phenotypical distribution *ex vivo* of these populations. Further gating analysis is shown in the corresponding figures. Manual data analysis was performed using the FlowJo software.
